# Mesenchymal stromal/stem cell-derived exosomes as a potential therapeutic approach to osteoarthritis combined with type 2 diabetes mellitus

**DOI:** 10.3389/fcell.2025.1549096

**Published:** 2025-03-27

**Authors:** Siyi Xie, Meiling Liu, Yajie Kong, Yiming Yang, Ruixue Chen, Yuzhong Wang, Shuxing Cao, Yongzhou Song

**Affiliations:** ^1^ Department of Orthopedics, The Second Hospital of Hebei Medical University, Shijiazhuang, Hebei, China; ^2^ Hebei Medical University-National University of Ireland Galway Stem Cell Research Center, Hebei Medical University, Shijiazhuang, Hebei, China; ^3^ Hebei Key Laboratory of Rare Disease, The Second Hospital of Hebei Medical University, Shijiazhuang, Hebei, China

**Keywords:** osteoarthritis, type 2 diabetes mellitus, diabetic osteoarthritis, mesenchymal stromal/stem cell-derived exosomes, microRNAs, engineered exosomes

## Abstract

Osteoarthritis (OA) and type 2 diabetes mellitus (T2DM) often coexist due to shared risk factors and high prevalence, but effective treatment methods are currently lacking. Mesenchymal stromal/stem cell-derived exosomes (MSC-Exos) have regenerative properties that can repair cartilage damage, lower blood sugar levels, and improve pancreatic β cell function, showing great potential in tissue repair. This review primarily explores the application of MSC-Exos in the treatment of OA and T2DM, the potential mechanisms of MSC-Exos, and the therapeutic strategies of engineered exosomes. Although MSC-Exo therapy shows promising therapeutic potential, further research is needed to validate its safety and feasibility.

## Graphical Abstract

**Figure:**
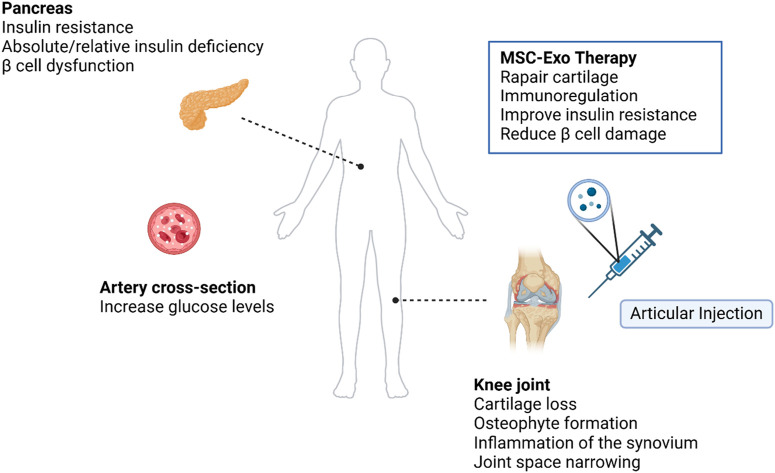
The graphical abstract was created with BioRender (www.biorender.com). Exploring the feasibility of intra-articular MSC-Exo therapy for symptoms in patients with T2DM combined with OA.

## 1 Introduction

OA is a chronic age-related degenerative joint disease that affects the entire synovial joint. It is characterized by structural damage to the articular hyaline cartilage, degeneration of subchondral bone, and changes in synovial tissue such as hypertrophy and increased vascularization (Loeser et al., 2012). T2DM is a metabolic disease caused by a lack of insulin or the body’s inability to use insulin effectively. T2DM is characterized by high blood sugar, and prolonged high blood sugar can lead to osmotic and oxidative stress, which can cause damage to various tissues, including bones, joints, kidneys, and the nervous system (Javeed and Matveyenko, 2018). Studies have shown that T2DM is an independent risk factor for OA (Schett et al., 2013). In T2DM, the interaction of joint degeneration and metabolic disorder can aggravate cartilage destruction and synovial inflammation, and OA and T2DM coexist to form diabetic OA (Eitner and Wildemann, 2021; Yang et al., 2024).

**FIGURE 1:**
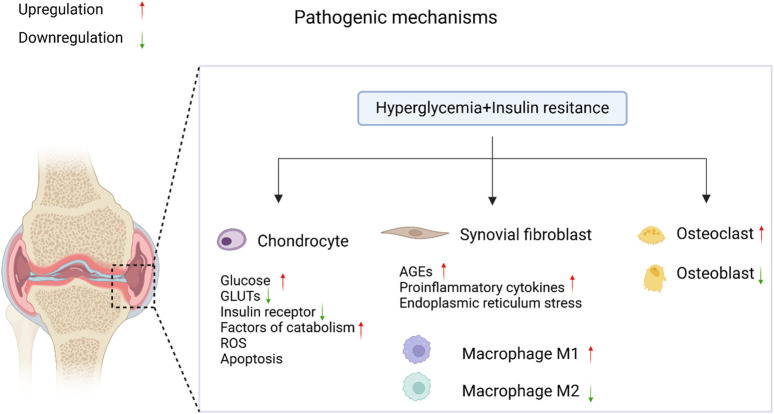
The pathological mechanisms of diabetic OA. In the coexistence of T2DM and OA, elevated blood glucose levels and insulin resistance accelerate the progression of diabetic OA through the regulation of signaling pathways. It was created with BioRender (www.biorender.com).

OA is a complex disease that affects multiple parts of the joint, including articular cartilage, subchondral bone, and synovium, and is accompanied by chronic inflammation (Courties and Sellam, 2016). Articular cartilage is composed of chondrocytes and the extracellular matrix (ECM), and its primary function is to absorb mechanical stress between bones. In OA, cartilage damage, synovial inflammation, osteophyte formation, and changes in joint morphology increase joint pressure, leading to the release of more pro-inflammatory mediators by chondrocytes, including cytokines [such as interleukin-1β (IL-1β) and tumor necrosis factor-alpha (TNF-α)], reactive oxygen species (ROS), and advanced glycation end-products (AGEs). This, in turn, triggers an increase in proteolytic enzymes [such as matrix metalloproteinases (MMPs) and aggrecanases with thrombospondin motifs (ADAMTS)], ultimately resulting in the degradation of the cartilage matrix. As shown in Figure 1, T2DM has a pathogenic effect on OA through two major pathways: (1) chronic hyperglycemia, which promotes oxidative stress, bolsters pro-inflammatory cytokines and AGEs production in joint tissues but also decreases the chondrogenic differentiation potential of MSCs, thereby further decreasing the already impaired cartilage repair in OA; and (2) insulin resistance (IR), which executes its effects both locally and also through low-grade inflammation systemically (Veronese et al., 2019). Chondrocyte damage and apoptosis might be induced due to leptin secretion from adipose tissue leading to the increased production of cytokine and MMPs (Courties and Sellam, 2016).

The main clinical treatment options for T2DM are glucose-lowering drugs and insulin therapy, but there is still a lack of a cure. Medications and other treatments can bring blood glucose levels as close to normal as possible, thereby delaying or preventing the onset of diabetes-related health problems (Su et al., 2023). While OA treatment includes surgery and medication. Surgery is aimed at repairing local cartilage damage, and medication is mainly anti-inflammatory, anti-catabolic, and symptomatic (Tejero et al., 2021; Madry, 2022). Current treatment methods can alleviate symptoms and reduce patient discomfort, but they do not provide a cure.

A study from the Osteoarthritis Initiative analyzed antidiabetic drugs and found that they may slow the progression of knee OA (Shirinsky and Shirinsky, 2017). Research indicates that the combination of metformin and cyclooxygenase-2 inhibitors might reduce the rate of joint replacement surgery in patients with OA and T2DM (Lu et al., 2018). However, Barnett et al. (2017) suggested no strong correlation between metformin treatment and OA development in diabetic patients, potentially due to confounding factors, diagnostic methods, and variations in dosage and duration of use. Additionally, studies show that long-term use of GLP-1 receptor agonists (GLP-1-RAs) can improve knee osteoarthritis (KOA) symptoms (Zhu et al., 2023), but both obese and non-obese diabetic patients using GLP-1-RAs may have an increased risk of developing KOA (Lavu et al., 2024). Given the controversy surrounding these treatments, exploring and developing new therapeutic strategies is particularly important.

Mesenchymal stromal/stem cells (MSCs) have demonstrated potential therapeutic effects in treating OA and T2DM. MSCs can promote cartilage repair and regeneration, reducing cartilage damage caused by arthritis, alleviating inflammation associated with arthritis and diabetes (Copp et al., 2023). There is growing evidence that many of the regenerative properties previously thought to be attributable to MSCs should be attributed to their secreted exosomes (Basu and Ludlow, 2016). In addition, MSC-Exos grafts have the advantages of being non-immunogenic, non-tumourigenic, and easy to store and transport compared to MSC therapy (Batrakova and Kim, 2015; Burger et al., 2015). Therefore, MSC-Exos have great potential for application in the field of tissue repair.

Therefore, this review aims to comprehensively assess the latest research progress in MSC-Exo therapy for diabetic OA, starting from relevant mechanisms and preclinical studies, summarizing current applications, analyzing feasibility and efficacy, exploring potential mechanisms, and providing directions for future research.

## 2 The mechanisms of OA and T2DM

OA and T2DM are two common diseases that frequently coexist due to their high prevalence and shared risk factors such as age, gender, ethnicity, and metabolic disorders (e.g., obesity, hypertension, and dyslipidemia) (Alenazi et al., 2023). Although their pathological mechanisms differ, they share common pathophysiological bases, including chronic inflammation, oxidative stress, and metabolic dysregulation.

### 2.1 The impact of T2DM on chondrocytes

Under hyperglycemic conditions, chondrocytes from OA patients exhibit multiple dysfunctions that collectively contribute to cartilage degradation and OA progression. Firstly, hyperglycemia prevents chondrocytes from effectively downregulating the expression of glucose transporter proteins (GLUTs), leading to excessive intracellular glucose accumulation (Sun et al., 2023). This hyperglycemic environment triggers the production of ROS (Rosa et al., 2009), whose accumulation further induces the release of inflammatory mediators (such as IL-1β and NF-κB), resulting in chondrocyte degradation and apoptosis, thereby compromising cartilage tissue integrity (Anderson et al., 2023). Additionally, OA chondrocytes in a high-glucose environment express significantly higher levels of MMPs, further exacerbating cartilage matrix degradation (Seow et al., 2024). Elevated glucose levels also inhibit the differentiation of MSCs into chondrocytes, impairing the regenerative capacity of damaged cartilage in OA (Courties and Sellam, 2016).

The effects of insulin on chondrocytes are concentration-dependent. Low concentrations of insulin may exert protective effects by promoting the synthesis of proteoglycans and type II collagen, while high concentrations of insulin may inhibit autophagy and induce the release of inflammatory mediators, thereby exacerbating cartilage degradation (Ribeiro et al., 2016). This dual effect may be related to differences in insulin receptor signaling at different concentrations. For example, low concentrations of insulin may promote chondrocyte anabolism by activating the Akt signaling pathway, while high concentrations of insulin may inhibit autophagy through the protein kinase B/mechanistic target of rapamycin (Akt/mTOR) pathway, leading to cartilage matrix degradation and enhanced inflammatory responses (Zhou et al., 2023).

Significant changes in the expression of transcription factors and protein kinases involved in the life cycle of chondrocytes have been observed in OA patients with diabetes. In chondrocytes from OA and diabetic patients, the microtubule-associated protein 1 light chain 3 (LC3) expression is reduced, and phosphorylating ribosomal S6 protein kinase (p-rpS6) expression is increased, which is attributed to autophagy defects (Ribeiro et al., 2016; Zhou et al., 2018). Autophagy is a key mechanism for maintaining chondrocyte homeostasis, and the inability to clear dysfunctional organelles and macromolecules in the absence of effective autophagy suggests a poor disease prognosis (de Figueroa et al., 2015). Higher protein kinase C (PKC) phosphorylation has been observed in MSCs maintained under high glucose conditions prior to chondrogenesis (Tsai et al., 2013b). Transforming growth factor-β (TGF-β)-stimulated Wingless (Wnt)-5a overexpression activates PKC-mediated mitogen-activated protein kinases, signaling the differentiation of chondrogenic cells into functional cells (Matta and Mobasheri, 2014). Vascular endothelial growth factor (VEGF) is hypothesized to mediate cartilage catabolism and endochondral ossification in osteoarthritis (Zupan et al., 2018). Since VEGF is significantly upregulated under hyperglycemic conditions (Zhang, 2018a), it is reasonable to assume that VEGF expression is higher in diabetic osteoarthritis compared to OA alone (Tsai et al., 2013a). SRY-box transcription factor 9 (SOX9), a chondroprotective factor typically downregulated in OA, is further reduced in diabetic osteoarthritis (Haseeb et al., 2021).

## 3 Comparison of traditional therapies, MSC therapy, and MSC-Exo therapy

MSC-Exos demonstrate significant advantages in the treatment of OA and T2DM. They enhance therapeutic effects by delivering active molecules such as microRNAs (miRNAs) and proteins to target cells (Ma et al., 2022), while also regulating inflammation, promoting tissue repair, and improving metabolism, showcasing potential through multi-target and multi-pathway mechanisms (Morente-López et al., 2022; Satyadev et al., 2023). In contrast, traditional therapies typically address only a single pathological aspect. For example, the long-term efficacy of anti-inflammatory drugs in alleviating OA symptoms has not been confirmed, and their use may lead to side effects (Rizzo et al., 2023); insulin therapy may induce hypoglycemia (Jadawji et al., 2018); and hypoglycemic agents carry risks such as weight changes and gastrointestinal discomfort, requiring frequent administration (Chawla et al., 2023).

Regarding MSC therapy, Phase I trials (Matas et al., 2024) have shown that MSCs can improve pain and joint function in OA patients, but larger-scale trials with control groups are needed for validation. Phase II studies (Lian et al., 2023) indicate that MSC treatment for T2DM is well-tolerated, though it may cause transient fever, hypoglycemia, fatigue, decreased lymphocyte levels, and increased inflammatory factors post-infusion. Despite their immunomodulatory and tissue-regenerative capabilities, MSCs still face challenges such as unstable cell sources, potential tumorigenic risks, infusion-related toxicity (Jeong et al., 2011; Fennema et al., 2017; Ma et al., 2022; Chawla et al., 2023), and negative effects on joints in metabolic mild OA (Warmink et al., 2023).

In comparison, MSC-Exos, being cell-free, avoid risks of immune rejection and abnormal cell proliferation (Lee, 2018). Due to the lower expression of surface proteins (e.g., major histocompatibility complex), MSC-Exos exhibit lower immunogenicity than their parent cells (Murphy et al., 2019). Engineered exosome strategies (Komuro et al., 2022) can further enhance their bioactivity and bioavailability. Therefore, MSC-Exos not only inherit the regenerative properties of MSCs (Basu and Ludlow, 2016) but also demonstrate superior targeting, stability, and broader mechanisms of action, offering more significant advantages over traditional therapies and MSCs.

## 4 The biosynthesis, isolation, characterization, and functional applications of exosomes

### 4.1 The biosynthesis and composition of exosomes

Extracellular vesicles (EVs) are present in bodily fluids, secreted by cells, and possess a membranous structure. The particles can be categorised into four divisions based on their size: exosomes (30–150 nm), microvesicles (100–1,000 nm), apoptotic bodies (50–5,000 nm, formed after cell apoptosis), and oncosomes (1–10 μm), recently identified and detected in cancer cells (Kou et al., 2022). Abundant studies indicate that exosomes and microvesicles play a pivotal role in the functioning of EVs in various physiological and pathological processes (Jeppesen et al., 2019).

Exosomes are tiny vesicles that have been discovered to have a density ranging from 1.11 to 1.19 g/mL. When observed under an electron microscope, they exhibit a characteristic disk-like structure and a flat spherical shape (Colombo et al., 2014). Various types of cells present in different bodily fluids and cell supernatants have the ability to release exosomes in both normal and pathological circumstances (Kou et al., 2022).

**FIGURE 2:**
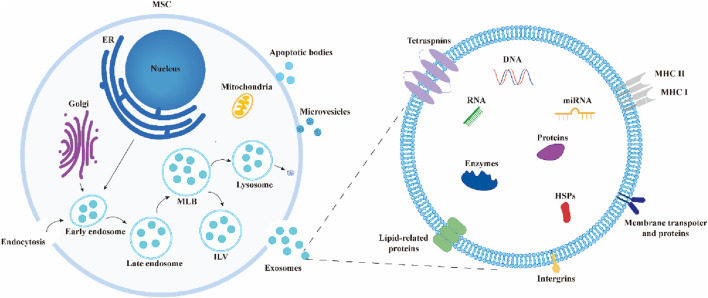
Biosynthesis and composition of exosomes. During the formation of exosomes, early endosomes encapsulate extracellular components and membrane proteins, forming multivesicular bodies (MVBs) that contain intraluminal vesicles (ILVs). These MVBs ultimately release exosomes through fusion with the plasma membrane or undergo degradation by fusing with autophagosomes or lysosomes. Exosomes are composed of lipids, proteins, nucleic acids, enzymes, and other components.

Exosomes often arise from the endosomal system by processes such as internal budding and invagination of the plasma membrane. This is commonly followed by the production of multivesicular bodies (MVBs) (Kalluri and LeBleu, 2020). Endosomes are initially formed through the process of internal budding of the plasma membrane. This results in the creation of both early endosomes and late endosomes. Following a series of consecutive or dual inward folding of the plasma membrane, intraluminal vesicles (ILVs) are generated within MVBs, which serve as progenitors to exosomes. They can undergo degradation and be released into the cytoplasm through fusion with autophagosomes or lysosomes. Alternatively, they can be released into EVs through fusion with the plasma membrane, including ILVs, which leads to the production of exosomes (Piper and Katzmann, 2007) (Figure 2).

### 4.2 The isolation, identification, and characterization of exosomes

Exosome isolation methods each have their own advantages and disadvantages. Ultracentrifugation is the “gold standard” for exosome isolation, with approximately 80% of studies using this technique (Ludwig et al., 2018). Its advantages include no need for complex sample preparation and low cost, but it is time-consuming and offers moderate purity. Ultrafiltration is fast and high-throughput but may damage exosomes due to shear stress or cause loss due to membrane clogging (Li et al., 2017), reducing yield and prolonging processing time (Dehghani et al., 2020). Precipitation offers high yield but lower purity and is often combined with other methods (Hammerschmidt et al., 2016). Immunoaffinity capture achieves high specificity and preserves biological activity but is limited by antibody availability, small sample capacity, and long incubation times (Yang et al., 2020). Size-exclusion chromatography (SEC) is gentle and efficient, maintaining vesicle integrity (Konoshenko et al., 2018), but requires pre-processing and results in low sample concentration, often needing additional enrichment steps. Commercial kits and emerging technologies (e.g., microfluidics and tangential flow filtration) significantly improve yield and purity (Abreu et al., 2022).

**FIGURE 3:**
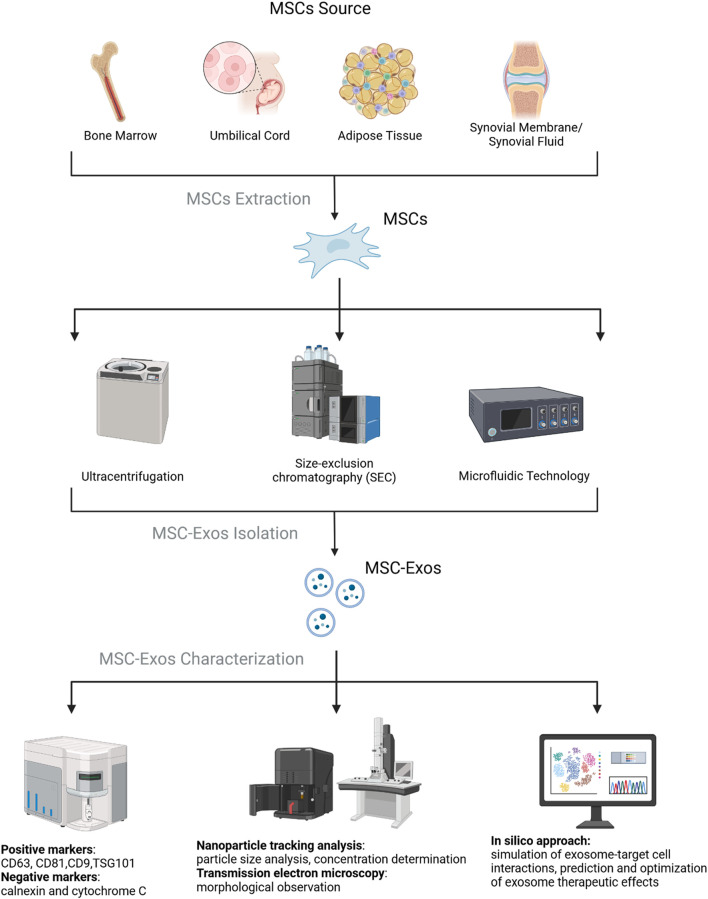
Cell source, isolation, and characterization of MSC-Exos. It was created with BioRender (www.biorender.com).

After isolation, exosomes are often validated using transmission electron microscopy (TEM) (Lai et al., 2022). Exosomes are rich in bioactive molecules, including proteins (e.g., receptors, enzymes, transcription factors), nucleic acids (e.g., DNA, RNA), and lipids (Valadi et al., 2007; Simpson et al., 2009). Most exosomes carry conserved proteins, such as tetraspanins (CD81, CD63, CD9), heat shock proteins (HSP60, HSP70, HSP90), ALIX, and TSG101, which are widely used as exosome biomarkers (Zhang et al., 2019). Studies show that MSC-Exos consistently express CD9, CD63, CD81, and TSG101 but do not express calnexin and cytochrome C, making these markers commonly used for MSC-Exos characterization (Maumus et al., 2020) (Figure 3).

### 4.3 The sources, dose and administration routes of MSC-Exos

MSCs can be isolated from a variety of tissues, including adipose tissue, bone marrow, umbilical cord, synovium, or infrapatellar fat pad. The phenotype and function of MSC-Exos may vary depending on the tissue origin of the MSCs (Börger et al., 2017). Research has shown that MSC-Exos derived from different human tissues exhibit distinct biological properties and therapeutic effects *in vivo* (Wang et al., 2017). Therefore, differences in the tissue origin of MSC-Exos may influence their therapeutic efficacy.

The delivery route of MSC-Exos can influence therapeutic efficacy, and different routes are used depending on the disease. For example, intra-articular injection of bone marrow mesenchymal stem cell-derived exosomes (BM-MSC-Exos) is used to treat OA patients (Do et al., 2020), nebulized umbilical cord mesenchymal stem cell-derived exosomes (UC-MSC-Exos) is administered to treat severe COVID-19 patients (Chu et al., 2022), and intrathecal injection of UC-MSC-Exos is applied for patients with complete subacute spinal cord injury (Akhlaghpasand et al., 2024). However, the standardization of MSC-Exos dosing remains challenging, as different studies use varying units of measurement: some studies use micrograms by weight, others use particle counts, while some simply reference the number of MSCs used to generate the MSC-Exos (Lotfy et al., 2023). This heterogeneity in dosing metrics makes direct comparison of results across studies difficult and highlights the urgent need to establish a unified dosing standard.

Given these issues, future preclinical studies must systematically investigate critical parameters such as the selection of MSC-Exo sources, determination of the minimum effective dose, and optimization of administration routes to develop disease-specific individualized treatment protocols. These studies will provide essential theoretical foundations and practical guidance for the clinical application of MSC-Exos.

### 4.4 The biological functions of MSC-Exos

The proteins included in the MSC-Exos belong to a distinct protein subclass that governs their distinctive biological roles (Kalluri and LeBleu, 2020). Simultaneously, the enclosed mRNA and miRNA within MSC-Exos serve as the fundamental components for their functionality (Qiu et al., 2018). MSC-Exos facilitate information transfer and communication with target cells by delivering a variety of molecules, including cytokines, growth factors, signaling lipids, mRNAs, and regulatory miRNAs, thereby altering the activities and functions of the target cells (Phinney and Pittenger, 2017). Studies have shown that MSC-Exos possess the ability to protect molecules from degradation and facilitate their efficient uptake into cells through endocytosis (Bagno et al., 2018). Additionally, MSC-Exos can serve as an ideal carrier system for transiently regulating specific biological processes in target cells (Rao et al., 2022), with the advantage of enabling cell-type-specific targeted delivery through surface modifications (Yang et al., 2018). MSC-Exos which not only possess immunomodulatory and tissue regeneration capabilities but also demonstrate certain advantages in immune therapy (Wu et al., 2018). These regenerative properties make MSC-Exos a promising tool for cell-free therapy in the treatment of various diseases.

## 5 Exosomal miRNAs: emerging roles in disease mechanisms and therapeutic potential

Exosomal miRNAs can be absorbed by nearby or distant cells, exerting biological effects by inhibiting target genes in recipient cells (Qin et al., 2019). These miRNAs are stable in circulation and are secreted by donor cells to act on distant recipient cells, regulating their gene expression (Broussard et al., 2016). miRNAs play a crucial role in gene expression regulation, and their functions can be divided into two categories:one is the classic negative regulatory function, where they inhibit gene expression by targeting mRNA; the other is a newly discovered function, such as acting as ligands to bind toll-like receptors (TLRs) and activate immune cells (Zhang et al., 2015). For example, exosomal miR-21 and miR-29a not only target mRNA but also trigger immune responses by binding to TLRs (Fabbri et al., 2012), a discovery that has opened new directions for miRNA functional research.

The expression levels of exosomal miRNAs can change with physiological conditions (Skog et al., 2008), and their surface proteins can also reflect their cellular or tissue origins (Mathivanan et al., 2010). Additionally, differences in the quantity and composition of exosomal miRNAs between diseased individuals and healthy individuals make them potential non-invasive biomarkers for early disease diagnosis and monitoring. Furthermore, exosomes can not only transport endogenous miRNAs but also sort and transfer exogenous miRNAs (Pegtel et al., 2010), a mechanism similar to that of endogenous miRNAs, providing new perspectives for studying intercellular communication.

In exosome functional studies, researchers use diverse technical approaches for comprehensive analysis. Proteomic analysis employs tandem mass spectrometry (labeling and label-free techniques), while miRNA profiling relies on high-throughput sequencing (Bi et al., 2022). Functional effects are explored through meta-analysis integrating miRNA and proteomics data. Bioinformatics tools like miRPathDB translate miRNA profiles into target gene information for pathway analysis (Ding et al., 2024). Validation of exosomal proteins and miRNAs is performed using Western blot and RT-qPCR. These integrated technologies provide a robust framework for exosome research.

In disease research, the genetic mechanisms of T2DM and OA are not yet fully understood, making the identification of new therapeutic targets crucial for early diagnosis and specific intervention. In recent years, computer simulation methods have shown great potential in disease research, such as predicting disease-related molecular functions (Raghav and Mann, 2024), lncRNA-miRNA interactions (Wang et al., 2022), and associations between metabolites (Sun et al., 2022). Comprehensive analysis based on transcriptomic data can identify differentially expressed genes (DEGs) and reveal their biological functions in diseases (Song and Yu, 2024). For example, the miR-29 family (including miR-29a, miR-29b, and miR-29c) plays an important role in the pathogenesis of T2DM and OA (Marttila et al., 2021; Mao et al., 2024).

Combining exosome analysis with disease research holds promise for discovering new therapeutic targets. Moreover, predicting biomarkers not only saves research time but also has potential clinical value. Research based on miRNAs provides new insights for disease diagnosis and treatment, and future studies could further explore their potential applications in precision medicine.

## 6 Key signaling pathways in OA and T2DM

### 6.1 PI3K/AKT/mTOR signaling pathway

The phosphoinositide 3-kinase (PI3K)/Akt/mTOR signaling pathway can be activated by various molecules, including insulin, glucose, growth factors, and cytokines (Engelman et al., 2006), playing a crucial role in OA and T2DM. Intervention strategies for OA based on this pathway can be divided into two categories: (1) inhibiting the pathway to restore cartilage homeostasis, enhance autophagy, and reduce inflammation, thereby alleviating joint damage; and (2) activating the pathway to promote chondrocyte proliferation and reduce apoptosis, exerting anti-arthritic effects (Sun et al., 2020). Under normal conditions, the PI3K/Akt pathway regulates metabolism and function, but its abnormal activation (e.g., overexpression or mutation) may lead to diseases such as obesity and cancer (Huang et al., 2018). The role of the mTOR pathway is dual, as it can either counteract or promote diabetes, depending on the cell type and physiological context (Jurca et al., 2023).

Insulin regulates inflammation, metabolism, and immune responses through the PI3K/Akt/mTOR pathway. In OA, insulin activates the PI3K/Akt/mTOR pathway, inhibits autophagy, and exacerbates cartilage degradation (Qiao et al., 2020). In immune regulation, insulin inhibits Toll-like receptor 4 (TLR4) and NF-κB through PI3K/Akt, exerting anti-inflammatory effects (Zhang et al., 2016), but it may also exacerbate inflammation by promoting Th17 differentiation and suppressing Treg function (Shi et al., 2019). In T Cells, insulin enhances glucose uptake and protein synthesis through the PI3K-Akt-mTOR pathway, promoting T Cell activation (Stentz and Kitabchi, 2003). Additionally, insulin influences immune responses by regulating macrophage polarization (M1 to M2 transition) and neutrophil function (Klauder et al., 2020). In bone metabolism, insulin promotes osteoblast differentiation and bone formation through PI3K/AKT/mTOR, while also promoting osteoclastogenesis via extracellular signal-regulated kinase 1/2 (ERK1/2) (Xian et al., 2012; Oh and Lee, 2017). mTOR complex 1 (mTORC1) plays a dual role in osteoblast and osteoclast differentiation, as its overactivation may inhibit osteoclastogenesis, while its inhibition may promote osteoclast differentiation (Hiraiwa et al., 2019).

MSC-Exos regulate the PI3K/Akt/mTOR pathway by delivering miRNAs and other bioactive molecules, demonstrating significant immunomodulatory and therapeutic potential. For example, MSC-Exos promote M2 macrophage polarization by delivering miRNAs such as miR-122-5p and miR-148a-3p (Li K. et al., 2022); infrapatellar fat pad mesenchymal stem cell-derived exosomes (IPFP-MSC-Exos) inhibit the mTOR autophagy pathway via miR-100-5p, maintaining cartilage homeostasis (Wu et al., 2019); synovial mesenchymal stem cell-derived exosomes (SMSC-Exos) carrying miR-485-3p alleviate OA cartilage damage by targeting the NRP1-mediated PI3K/Akt pathway (Qiu et al., 2024). Furthermore, BM-MSC-Exos reduce IR and obesity in mice through the PI3K/AKT pathway (Shi et al., 2023). Engineering approaches can enhance exosome efficacy. For instance, Wu et al. (2024) activated the PI3K/Akt pathway in infrapatellar fat pad-derived mesenchymal stem cell (IPFP-MSCs) through TNF-α pretreatment, upregulating autophagy-related protein 16 like 1 (ATG16L1) and promoting the secretion of low-density lipoprotein receptor-related protein 1 (LRP1)-enriched exosomes, effectively preventing OA cartilage damage.

### 6.2 NLRP3 inflammasome pathway

The formation of the NOD-like receptor family pyrin domain-containing 3 (NLRP3) inflammasome involves activation, assembly, and regulation. Its core mechanism involves the interaction of NLRP3 as a “receptor” and pro-Caspase-1 as an “effector.” Upon assembly, the inflammasome activates pro-Caspase-1, generating active Caspase-1, which processes pro-IL-1β and pro-IL-18 into mature IL-1β and IL-18, amplifying the inflammatory response. Simultaneously, NLRP3 inflammasome activation induces pyroptosis, further exacerbating inflammatory cell death (Broz, 2019).

In OA, pro-inflammatory factors such as IL-1β, IL-18, and TNF-α accelerate disease progression by increasing cartilage ECM degradation (Shi et al., 2019). Macrophages, the primary cells regulating OA inflammation, activate the NLRP3 inflammasome (Sanchez-Lopez et al., 2019), releasing various pro-inflammatory factors (Zhang et al., 2020) and cytokines (e.g., IL-1β and IL-18), further exacerbating OA inflammation and cartilage destruction.

In T2DM, glycolipid metabolites (e.g., glucose and free fatty acids) activate the NLRP3 inflammasome through multiple pathways (Wang et al., 2020). Chronic hyperglycemia and saturated fatty acids (e.g., palmitate) activate the NLRP3 inflammasome, promoting the secretion of IL-1β and IL-18, which interfere with insulin signaling and lead to IR (Vandanmagsar et al., 2011). Additionally, islet amyloid polypeptide (IAPP) deposits activate the NLRP3 inflammasome by disrupting lysosomes, triggering inflammatory responses (Masters et al., 2010). Studies have shown that inhibiting NLRP3 inflammasome activation (e.g., through Caspase-1 inhibitors or gene knockout) can alleviate β-cell inflammation and improve IR (Qi et al., 2019b).

Mesenchymal stem cell-derived EVs (MSC-EVs) show potential in OA treatment by slowing disease progression, promoting chondrocyte proliferation and migration, and inhibiting chondrocyte apoptosis. EVs carrying miR-1208 inhibit inflammation by targeting methyltransferase-like 3 (METTL3) to reduce NLRP3 mRNA methylation (Zhou et al., 2022). Liu et al. (2023) loaded exogenous miR-223 into MSC-EVs via electroporation and modified the MSC-EVs surface with a collagen II-targeting peptide (WYRGRL) through genetic engineering, achieving more targeted and efficient RNA delivery to cartilage. These dual-engineered EVs significantly inhibit NLRP3 inflammasome activation and chondrocyte pyroptosis, providing a novel strategy for OA treatment.

Multiple signaling pathways have been shown to play roles in OA and T2DM progression, but further research is needed to identify specific common targets (e.g., miRNAs and target genes). MSC-Exos demonstrate broad prospects in modulating inflammation and promoting tissue repair, but their precise mechanisms require further elucidation. Future research should focus on exploring the intersections of these signaling pathways to develop more precise therapeutic approaches.

## 7 Potential role of MSC-Exos in the treatment of OA

### 7.1 Regulation of the ECM

The gradual degradation of cartilage matrix is the key pathology of OA, leading to structural damage of joints and subsequent injury. To stimulate the redeposition of cartilage ECM and preserve the structural integrity of cartilage, it is imperative to initiate repair mechanisms in chondrocytes and increase the expression of genes related to synthetic metabolism (Heard et al., 2015).

Several studies have shown that MSC-Exos help to maintain the equilibrium of the ECM. Zhang et al. (2018b) discovered that embryonic stem cell-derived MSC exosomes promoted the synthesis of important cartilage matrix components such as sulfated glycosaminoglycans and type II collagen, accelerating chondrocyte repair. Jammes et al. (2023) discovered that BM-MSC-Exos effectively enhanced the production of a matrix resembling transparency. This was achieved by controlling the amounts of collagen proteins, promoting the expression of proliferating cell nuclear antigen, and decreasing the synthesis of Htra1. Woo et al. (2020) showed that adipose-derived mesenchymal stem cell-derived exosomes (AD-MSC-Exos) effectively enhanced the expression of type II collagen in chondrocytes while reducing the expression of ADAMTS-5, MMP-1, MMP-3, and MMP-13, thereby alleviating cartilage matrix degradation in a monoiodoacetate-induced OA model.

Exosomes RNA has shown significant potential in promoting cartilage ECM repair. Chen et al. (2020) found that miR-136-5p from bone marrow-derived mesenchymal stem cells (BM-MSCs) upregulated the expression of type II collagen, aggrecan, and SOX9, while downregulating MMP-13 expression, thereby promoting chondrocyte migration. Xia et al. (2021) showed that BM-MSC-exosomal miR-125a-5p alleviated chondrocyte ECM degradation in post-traumatic OA by inhibiting E2F2. Zhou et al. (2022) discovered that UC-MSC-Exos suppressed the breakdown of ECM in a mouse model of OA by using miR-1208.

### 7.2 Effects on cartilage repair

In the inflammatory environment and conditions of KOA, chondrocyte metabolic homeostasis is disrupted, leading to cartilage remodeling characterized by enhanced glycolytic pathway, mitochondrial dysfunction, and chondrocyte senescence (Mobasheri et al., 2017). Chen et al. (2019a) found that supplementing mitochondrial-related proteins using MSC-Exos in degenerated cartilage restored mitochondrial dysfunction and oxidative stress damage, thus rescuing energy metabolism imbalance and promoting cartilage regeneration. Wu et al. (2019) discovered that IPFP-MSC-Exos suppressed chondrocyte apoptosis by inhibiting the mTOR signaling pathway targeted at chondrocytes, thereby regulating chondrocyte metabolism and promoting ECM regeneration. Qi et al. (2019a) pointed out that BM-MSC-Exos enhance the phosphorylation of Akt while decreasing the phosphorylation of ERK and p38, which leads to the inhibition of chondrocyte apoptosis produced by mitochondria.

Cartilage’s avascular nature and limited interchange of signaling chemicals, oxygen, and nutrients pose considerable difficulties to its self-repair capacities (Carballo et al., 2017). MSC-Exos have exhibited robust capacities in selectively influencing biological processes such as the growth and death of chondrocytes (Xiang et al., 2022). Another study confirmed that AD-MSC-Exos-derived exosomal miR-338-3p transplantation can inhibit chondrocyte inflammation and degradation by targeting Runt-related transcription factor 2 (RUNX2), while promoting chondrocyte proliferation (Li C. et al., 2022a). Li et al. (2021) revealed that exosomes derived from induced pluripotent stem cell-derived MSCs and synovial fluid mesenchymal stem cell-derived exosomes (SF-MSC-Exos) significantly promoted chondrocyte proliferation and migration.

### 7.3 Immunoregulation

The progression of OA is directly related to the extent of inflammatory infiltration. The secretion of inflammatory cytokines triggers immune responses that contribute to the development and advancement of OA. Several studies have demonstrated that MSC-Exos can regulate inflammation by decreasing the levels of pro-inflammatory cytokines and stimulating the secretion of anti-inflammatory cytokines (Hassanzadeh et al., 2023). Furthermore, synovial macrophages are the primary immune cells in the knee joint, MSC-Exos can inhibit macrophage recruitment.

Song et al. (2023) discovered that MSC-Exos can suppress macrophage ferroptosis through the activation of the GOT1/CCR2/Nrf2/HO-1 signaling pathway. As a result, they can effectively repair cartilage degradation in OA. Ragni et al. (2020) confirmed protective and anti-inflammatory activity against macrophages and chondrocytes in computer simulations, consistent with *in vitro* findings that the MSC secretome and its initiation inhibited chondrocyte catabolism and inflammatory markers as well as macrophage activation. Cosenza et al. (2018) studied the immunosuppressive impacts of microvesicles and exosomes produced from MSCs on T and B lymphocytes in laboratory settings and in models of delayed-type hypersensitivit and collagen-induced arthritis.

### 7.4 Anti-inflammatory response

In the pathogenesis of KOA, inflammatory responses play a crucial role. The key determinants in managing this condition seem to be the equilibrium between pro-inflammatory cytokines and anti-inflammatory cytokines, which have opposing influences. The former primarily include IL-1β, TNF-α, IL-6, IL-15, IL-17, and IL-18. Conversely, anti-inflammatory cytokines induced by TNF include IL-4, IL-10, IL-13, IL-37, among others.

Zhou et al. (2022) revealed that MSC-Exos effectively inhibit the activation of the NOD-like receptor family pyrin domain containing 3 (NLRP3) inflammasome in macrophages, resulting in reduced secretion of IL-1β and IL-18, thereby successfully alleviating OA. Wang et al. (2024) demonstrated that exosomes-shuttled lncRNA small nucleolar RNA host gene 7 (SNHG7) by BM-MSCs alleviates OA through targeting miR-485-5p/ferroptosis suppressor protein 1 (FSP1) axis-mediated chondrocytes ferroptosis and inflammation. Qiu et al. (2021) showed that SMSC-Exos containing miR-129-5p mitigate inflammation induced by IL-1β in OA by inhibiting high mobility group box 1 (HMGB1) release (Table 1).

**TABLE 1:** Potential role of MSC-Exos in the treatment of OA.

Source	Cargo	Model	Outcome	Ref
EMSC		Chondrocyte,Rat osteochondral defect model	Promote cartilage repair and regeneration, attend attenuateat apoptosis and modulate immune reactivity	Zhang (2018b)
BM-MSC		Chondrocyte	Enhance the production of a matrix resembling transparency	Jammes et al. (2023)
AD-MSC		Chondrocyte, MIA rat model, DMM mouse model	Promote the proliferation and migration of human OA chondrocytes, maintain the chondrocyte matrix	Woo et al. (2020)
BM-MSC	miR-136-5p	Chondrocyte, Post-traumatic OA mouse model	Reduce the degeneration of cartilage ECM	Chen et al. (2020)
BM-MSC	miR-125a-5p	Chondrocyte, Post-traumatic OA mouse model	Alleviate chondrocyte ECM degradation	Xia et al. (2021)
UC-MSC	miR-1208	Chondrocyte, DMM mouse model	Decrease pro-inflammatory factor secretion, decrease osteophyte production by inhibiting E2F2	Zhou et al. (2022)
IPFP-MSC	miR-100-5p	Chondrocyte, DMM mouse model	Enhance the level of autophagy in chondrocytes via inhibition of mTOR signaling pathway	Wu et al. (2019)
BM-MSC		Chondrocyte	Inhibit chondrocyte apoptosis	Qi (2019a)
AD-MSC	miR-338-3p	The murine chondroprogenitor cell line ATDC5	inhibit chondrocyte inflammation and degradation, promote chondrocyte proliferation	Li C. et al. (2022)
BM-MSC	circHIPK3/miR-124-3p	Chondrocyte	induce chondrocyte proliferation and migration, inhibit chondrocyte apoptosis	Li et al. (2021)
MSC		MC3T3-E1 cell, OVX mouse model	inhibit macrophage ferroptosis, repair cartilage degradation	Song et al. (2023)
AD-MSC		Macrophage, chondrocyte	inhibit both chondrocyte catabolic and inflammatory markers and macrophage activation	Ragni et al. (2020)
BM-MSC		T lymphocyte, DTH mouse model, CIA mouse model	Play an anti-inflammatory role on T and B lymphocytes, suppress inflammation	Cosenza et al. (2018)
BM-MSC	lncRNA	Chondrocyte	Repress inflammatory injury, oxidative stress and ferroptosis	Wang et al. (2024)
SMSC	MiR-129-5p	Chondrocyte	Decline the inflammatory response and apoptosis of chondrocytes	Qiu et al. (2021)

Abbreviation: MSC, mesenchymal stromal/stem cells; AD-MSC, Adipose-derived MSC; BM-MSC, bone marrow derived MSC; EMSC, embryonic MSC; IPFP-MSC, infrapatellar fat pad derived MSC; SMSC, synovial MSC; SF-MSC, synovial fluid derived MSC; UC-MSC, umbilical cord derived MSC; MIA, monosodium iodoacetate; ACLT, anterior cruciate ligament transection; DMM, destabilization of the medial meniscus; OVX, bilateral ovariectomy; DTH, delayed-type hypersensitivity; CIA, collagen-induced arthritis.

## 8 Potential role of MSC-Exos in the treatment of T2DM

### 8.1 Improvement of IR

Inflammation is a critical factor in the development of IR that is linked to T2DM associated with obesity. M1 macrophages located in adipose tissue release many substances, including TNF-α, IL-6, IL-1β, and monocyte chemotactic protein-1. These substances promote inflammation and initiate IIR in insulin-responsive cells such as adipocytes, skeletal muscle cells, and pancreatic cells (Olefsky and Glass, 2010; Tateya et al., 2013).

Su et al. (2019) reported that BM-MSC-Exos containing miR-29b regulates age-related IR by targeting sirtuin (SIRT)1 in adipocytes, muscle cells, and liver cells. Chen et al. (2021) found that UC-MSC-Exos enhance insulin sensitivity in human adipocytes by inhibiting the production of the adipokine leptin and increasing the mRNA expression of adiponectin, SIRT1, and insulin receptor substrate-1 (IRS-1). Shi et al. (2023) found that BM-MSC-Exos increased glucose uptake and improved IR in high-fat diet-fed mice and palmitic acid-treated 3T3-L1 adipocytes by activating the phosphoinositide 3-kinase (PI3K/Akt) signaling pathway and upregulating GLUT4 expression. Sun et al. (2018) demonstrated that intravenous administration of UC-MSC-Exos significantly reduced blood glucose levels and partially reversed IR in a rat model of T2DM induced by a high-fat diet and streptozotocin (STZ). The treatment with UC-MSC-Exos restored the phosphorylation of IRS-1 and Akt at tyrosine sites, thereby enhancing the expression and membrane translocation of GLUT-4 in muscle tissues. Additionally, UC-MSC-Exos promoted hepatic glycogen storage, contributing to the maintenance of glucose homeostasis. Furthermore, UC-MSC-Exos inhibited STZ-induced β cell apoptosis and restored insulin secretion function in T2DM.

### 8.2 Effects on pancreatic β cells

One major physiological barrier for patients with T2DM is inadequate insulin secretion, leading to sustained hyperglycemia. Prolonged high blood glucose levels can deplete insulin stores and lead to compensatory insulin secretion, which damages pancreatic β cells.

Studies have shown that umbilical cord-derived mesenchymal stem cells (UC-MSCs) can induce insulin-producing cells *in vitro* but do not differentiate into pancreatic β cells *in vivo*, mainly exerting their effects through paracrine actions in T2DM (Nagaishi et al., 2016). In a study by Sharma et al. (2021), treatment with UC-MSC-Exos via intravenous injection reduced hyperglycemia in T2DM mice, increased insulin production, and improved tissue structure. Analysis of pancreatic tissue samples revealed elevated expression of genes involved in pancreatic tissue regeneration pathways (Reg2, Reg3, and Amy2b). MiRNA analysis of MSC-Exos indicated potential promotion of pancreatic regeneration pathways, possibly through modulation of the Extl3-Reg-cyclinD1 pathway. These results suggest that UC-MSC-Exos have therapeutic potential in alleviating insulin deficiency by activating pancreatic regeneration. Additionally, Xia et al. (2024) demonstrated that MSC-Exos suppressed nuclear factor erythroid 2 related factor (NRF2)-mediated ferroptosis by delivering bioactive proteins to regulate the Akt/ERK signaling pathway, thereby improving the function and quantity of β cells. They modified the β cell targeting aptamer with polyethylene glycol on the membrane surface of exosomes, and the former mediated β cell targeting was more effective in islet protection compared to unmodified MSC-Exos (Table 2).

**TABLE 2:** Potential role of MSC-Exos in the treatment of T2DM.

Source	Cargo	Model	Outcome	Ref
BM-MSC	MiR-29b-3p	Hepatocyte	Modulate aging-related IR	Su et al. (2019)
UC-MSC		Adipocyte	Improve insulin sensitivity in insulin resistant human adipocytes	Chen et al. (2021)
BM-MSC		Mature 3T3-L1 adipocyte (palmitate), T2DM mouse model (STZ)	Increased glucose uptake and improve insulin resistance	Shi et al. (2023)
UC-MSC		L02 cell (palmitic acid), T2DM rat model (HFD + STZ)	Maintain glucose homeostasis, reverse insulin resistance, restore the insulin-secreting function	Sun et al. (2018)
UC-MSC		T2DM mouse model (HFD + STZ)	Reduce hyperglycemia, increase insulin production, improve tissue structure	Sharma et al. (2021)
UC-MSC		The high glucose-stimulated INS-1 cell line, T2DM mouse model (HFD + STZ)	Reduce random blood glucose levels, enhance glucose, insulin tolerance, increase insulin secretion	Xia et al. (2024)

Abbreviation: MSC, mesenchymal stromal/stem cells; BM-MSC, bone marrow derived MSC; UC-MSC, umbilical cord derived MSC; HFD, high-fat diet; STZ, streptozotocin.

## 9 Engineered MSC-Exos for therapeutic applications

**FIGURE 4:**
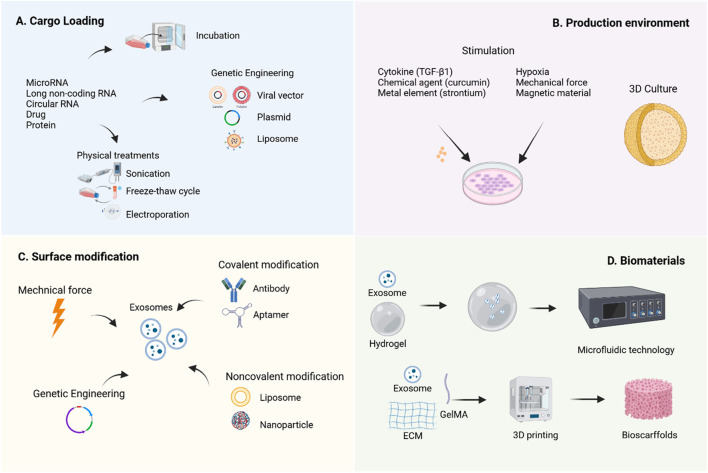
Engineering strategies for MSC-Exos. To enhance the therapeutic efficacy of MSC-Exos, various engineering strategies have been developed to improve their yield, bioactivity, and bioavailability. It was created with BioRender (www.biorender.com).

Engineering strategies aim to overcome the limitations of natural exosomes through various approaches (Komuro et al., 2022). Currently, the application of engineered MSC-Exos in OA treatment primarily focuses on content modification, membrane property optimization, and integration with biomaterials to enhance their bioactivity and bioavailability (Figure 4).

### 9.1 Cell modification

Cell modification is a method to indirectly alter the contents or membrane properties of exosomes before isolation, serving as a rapid response mechanism of parental cells to environmental stimuli (Lai et al., 2014). The simplest approach for cargo loading prior to isolation involves co-incubating the target cargo with exosome-secreting cells, allowing the cargo to diffuse into the exosomes via a concentration gradient (Oskouie et al., 2018). Additionally, transfection techniques can be employed to introduce specific plasmids into cells, enabling the ectopic expression of target biomolecules within exosomes. Physical methods such as sonication, electroporation, extrusion, freeze-thaw cycles, surfactant treatment, and dialysis are also widely utilized (Luan et al., 2017). Research indicates that natural exosomes enter recipient cells through free diffusion and are subsequently internalized randomly (Lai et al., 2014).

The modification of MSC-Exos content primarily relies on the overexpression of specific non-coding RNAs to enrich therapeutic molecules. Studies have shown that miRNAs play a crucial regulatory role in exosome-mediated cartilage repair (Foo et al., 2021). Currently, researchers commonly use transfection tools such as viral vectors (Chen, 2019b), plasmids (Li F. et al., 2022), and liposomes (Liu, 2021b) to genetically engineer parent cells, thereby obtaining modified exosomes carrying specific molecules. Tao et al. (2017) found that miR-140-5p-enriched exosomes secreted by synovial mesenchymal stem cells (SMSCs) through transfection technology significantly promoted chondrocyte proliferation and migration, effectively alleviated symptoms in a rat OA model, and did not compromise ECM secretion. Morente-López et al. (2022) reported that EVs derived from UC-MSCs with lentivirus-induced miR-21 inhibition effectively reduced the levels of chemokines and cytokines in the serum of OA animals and decreased the senescence-associated secretory phenotype.

### 9.2 Production environment

Modulating the production environment of MSC-Exos for engineering transformation has emerged as a highly promising research direction, encompassing two major aspects: biochemical factors and biophysical factors. In terms of biochemical regulation, TNF-α pretreatment significantly enhances IPFP-MSC-Exos and improves their therapeutic efficacy for OA (Wu et al., 2024). MSC-Exos derived from curcumin-induced adipose tissue exhibit enhanced antioxidant stress and anti-chondrocyte apoptosis capabilities, thereby improving their therapeutic efficacy for OA (Xu et al., 2022). Additionally, exosomes produced by strontium-substituted calcium silicate-treated bone marrow mesenchymal stem cells demonstrate a unique dual regulatory function, promoting both osteogenesis and angiogenesis (Liu, 2021a). Regarding biophysical regulation, studies have shown that hypoxia-treated MSCs not only increase exosome yield but also enhance their ability to promote cell proliferation and differentiation (Pulido-Escribano et al., 2022). Notably, MSC-EVs generated under hypoxic conditions (Hypo-EVs) exhibit superior efficacy in OA cartilage repair compared to exosomes produced under normoxic conditions (Rong et al., 2021). Furthermore, Yan and Wu (2019) demonstrated that UC-MSC-Exos cultured in a 3D hollow fiber bioreactor not only show increased yield but also significantly enhanced cartilage-protective effects.

### 9.3 Direct exosome engineering

Direct exosome engineering refers to the post-purification process of modifying exosomes through physical, chemical, or genetic methods to enhance their cargo or membrane properties, thereby improving their targeting capabilities and/or therapeutic efficacy. Some modification techniques originally applied to parent cells can also be directly utilized on exosomes (Zhu et al., 2024).

Zhao et al. (2023) discovered that cartilage-affinity peptide (CAP)-conjugated exosomes derived from subcutaneous fat MSCs could specifically deliver miR-199a-3p to target cells and deep joint tissues, significantly impacting OA progression. Zhuang et al. (2023) developed superparamagnetic iron oxide nanoparticle (SPION)-modified exosomes to load quercetin, leveraging magnetic force (MF) to enhance quercetin’s water solubility and active targeting ability, thereby improving islet protection. These findings serve as valuable references, offering new strategies to enhance the therapeutic efficiency of MSC-EVs through direct modification of isolated EVs. Additionally, Luo et al. (2019) modified exosomes with aptamers via Schiff base reactions between aldehydes and amino groups, promoting bone repair by enhancing the targeting ability of BM-MSCs. A lipid-based strategy involving the combination of antagomir-188-loaded liposomes with exosomes from cells engineered with the CXC motif chemokine receptor 4 gene was employed to prevent the shift of BM-MSCs’ osteogenic differentiation toward adipogenesis, without requiring covalent modification (Hu et al., 2021). The underlying mechanism of non-covalent methods involves electrostatic interactions between the cargo and exosomes.

### 9.4 MSC-exos combined with biomaterials

The suboptimal therapeutic efficacy of OA is partly attributed to the inefficient drug delivery within the knee joint. By combining with biomaterials, MSC-Exos can achieve effective retention at pathological sites, improve drug delivery pathways, and thereby significantly enhance therapeutic outcomes (You et al., 2023).

Hydrogels, owing to their injectability and cross-linking capability under ultraviolet light, have become ideal biomaterials for tissue engineering. Their raw materials include natural polymers (such as polysaccharides like hyaluronic acid, alginate, and chitosan, as well as proteins like collagen, gelatin, and silk fibroin), synthetic polymers (such as polyglycolic acid, polycaprolactone, and polylactic acid), and composite materials (Bahraminasab et al., 2021). Research by Pang et al. (2023) demonstrated that gelatin methacryloyl hydrogel (GelMA) prolongs the release of MSC-Exos and significantly enhances their therapeutic efficacy against OA. Furthermore, advanced manufacturing methods such as electrospinning, 3D printing, and microfluidic technology have facilitated the development of nanogel/microgel porous scaffolds (Pishavar et al., 2021), further enriching their synergistic effects with exosomes. For instance, Yang et al. (Yang et al., 2023) developed an enzyme-responsive smart hydrogel using microfluidic chips by combining neovascularized bone matrix metalloproteinase-1, self-assembling hydrogels, and exosomes to synthesize injectable microgels. This hydrogel can specifically recognize neovascularization and precisely release exosomes in a spatiotemporal manner, promoting angiogenesis and osteogenesis.

Multidimensional modification strategies not only optimize the functional properties of exosomes but also open up broader prospects for their application in disease treatment. For example, Chen et al. (2024) utilized CRISPR/Cas9 technology to develop a targeted gene editing tool for fibroblast growth factor FGF18, delivered via CAP-conjugated hybrid exosomes (CAP/FGF18-hyEXO). This approach effectively activated the FGF18 gene in OA chondrocytes at the genomic level *in vivo*. The study confirmed that this strategy synergistically promotes cartilage regeneration, reduces inflammation, and prevents ECM degradation both *in vitro* and *in vivo*, demonstrating significant potential for clinical translation.

## 10 Discussion

Exosome-based cell-free approaches have shown promising results in the treatment of OA and T2DM. However, research on using MSC-Exos to treat diabetic OA remains limited. In studies on diabetic complications, Jin et al. (2019) demonstrated that AD-MSC-Exos alleviates diabetic nephropathy by promoting podocyte autophagy flux and inhibiting apoptosis. Hu et al. (2023) showed that hypoxia-preconditioned ADSC-Exos embedded in hydrogels promote angiogenesis and accelerate diabetic wound healing. Cao et al. (2021) proved that MSC-Exos inhibit endothelial-mesenchymal transition and tube formation in diabetic retinopathy. These studies provide valuable references for exosome-based therapies targeting T2DM complicated by OA.

The biological functions of MSC-Exos vary depending on their sources (Börger et al., 2017). Exploring the characterization of MSC-Exos across different subpopulations and accurately determining their cargo content is crucial, as it may significantly alter their impact on target tissues (Forsberg et al., 2020). In both preclinical and clinical studies, the specific minimum effective dose of MSC-Exos has not yet been determined. The most commonly used route in preclinical studies is intravenous injection (Hassanzadeh et al., 2021), along with intraperitoneal and subcutaneous injections. For OA treatment, intra-articular injection is employed (Do et al., 2020). There is an urgent need to standardize the parental MSC source, therapeutic dose, and administration routes for MSC-Exos products.

The application of exosomes as biologics in clinical settings faces multiple challenges, including standardization, safety, and quality control issues, such as the lack of standardized methods for collection and isolation, the inherent heterogeneity of exosomes, and contamination from exogenous sources. To ensure the safety and efficacy of clinical-grade exosome preparations, it is essential to adhere to Good Manufacturing Practice (GMP) protocols and use serum-free media to avoid animal-derived contamination. By studying the mechanisms of action of exosomes, regulating their key active components, and employing bioengineering techniques to modify exosome phenotypes or contents, therapeutic efficacy can be enhanced while reducing adverse effects (Ferreira et al., 2022). Additionally, screening exosome biomarkers (e.g., surface receptors) and optimizing isolation and purification methods can help obtain more homogeneous and potent exosome populations (Chen et al., 2022). In the future, a deeper understanding of the mechanisms of action, along with the establishment of standardized production processes and quality control strategies, will further unlock the potential of exosomes in clinical applications.

Natural exosomes have limitations such as insufficient secretion, poor targeting, short retention time, and heterogeneity (Zhu et al., 2024), which hinder their use in large-scale clinical trials. To achieve therapeutic effects, MSC-Exos need to carry sufficient doses of bioactive factors (such as proteins or miRNAs) with functional activity to effectively trigger biological responses in target cells (Toh et al., 2018). For example, miRNA content can be enriched through methods such as endogenous or exogenous loading. Additionally, modifying exosomes using bioengineering techniques can further enhance their bioactivity and therapeutic efficacy. However, it is important to note that while the design of engineered exosomes holds unlimited potential, we must also consider issues such as their loading efficiency, differentiation from natural exosomes, biocompatibility when combined with biomaterials, and potential adverse effects that may arise from engineered exosome therapy.

## 11 Conclusion

MSC-Exo therapy represents a highly promising cell-free therapeutic approach. The application of bioengineering technologies has significantly enhanced the therapeutic efficacy of MSC-Exos. Further research is needed to elucidate the mechanisms underlying diabetic OA, establish standardized criteria for evaluating therapeutic effects and safety, optimize engineered exosome treatment strategies, and accelerate the clinical translation of MSC-Exos.

## Glossary

ADAMTS: Aggrecanases with thrombospondin motifs
AD-MSC-Exos: Adipose-derived mesenchymal stem cell-derived exosomes
AGEs: Advanced glycation end-products
Akt: Protein kinase B
BM-MSCs: Bone marrow-derived mesenchymal stem cells
BM-MSC-Exos: Bone marrow mesenchymal stem cell-derived exosomes
CAP: Chondrocyte-affinity peptide
ECM: Extracellular matrix
EVs: Extracellular vesicles
GLP-1-RAs: Glucagon-like peptide-1 receptor agonists
GLUT: Glucose transporter protein
ILVs: Intraluminal vesicles
IPFP-MSC-Exos: Infrapatellar fat pad mesenchymal stem cell-derived exosomes
IR: Insulin resistance
IRS-1: Insulin receptor substrate-1
KOA: Knee osteoarthritis
miRNAs: microRNAs
MMPs: Matrix metalloproteinases
MSCs: Mesenchymal stromal/stem cells
MSC-Exos: Mesenchymal stromal/stem cell-derived exosomes
mTOR: Mechanistic target of rapamycin
MVBs: Multivesicular bodies
NLRP3: NOD-like receptor family pyrin domain containing 3
OA: Osteoarthritis
PI3K: Phosphoinositide 3-kinase
PKC: Protein kinase C
ROS: Reactive oxygen species
SIRT: Sirtuin
SMSC-Exos: Synovial mesenchymal stem cell-derived exosomes
SOX9: SRY-box transcription factor 9
STZ: Streptozotocin
TNF: Tumor necrosis factor
T2DM: Type 2 diabetes mellitus
UC-MSCs: Umbilical cord-derived mesenchymal stem cells
UC-MSC-Exos: Umbilical cord mesenchymal stem cell-derived exosomes
VEGF: Vascular endothelial growth factor.
